# Clinical and Computerized Volumetric Analysis of Posterior Fossa Decompression for Space-Occupying Cerebellar Infarction

**DOI:** 10.3389/fneur.2022.840212

**Published:** 2022-05-12

**Authors:** Eric Goulin Lippi Fernandes, Sami Ridwan, Isabell Greeve, Wolf-Rüdiger Schäbitz, Alexander Grote, Matthias Simon

**Affiliations:** ^1^Department of Neurosurgery, Evangelisches Klinikum Bethel, University Hospital OWL, University Bielefeld, Campus Bielefeld-Bethel, Bielefeld, Germany; ^2^Department of Neurosurgery, Klinikum Ibbenbüren, Ibbenbüren, Germany; ^3^Department of Neurology, Evangelisches Klinikum Bethel, University Hospital OWL, University Bielefeld, Campus Bielefeld-Bethel, Bielefeld, Germany

**Keywords:** cerebellar infarction, surgical management, decompression, posterior cranial fossa decompression, space-occupying cerebellar infarction, volumetric analysis, clinical analysis, outcome

## Abstract

**Background and Purpose:**

Surgical decompression of the posterior fossa is often performed in cases with a space-occupying cerebellar infarction to prevent coma and death. In this study, we analyzed our institutional experience with this condition. We specifically attempted to address timing issues and investigated the role of cerebellar necrosectomy using imaging data and conducting volumetric analyses.

**Methods:**

We retrospectively studied pertinent clinical and imaging data, including computerized volumetric analyses (preoperative/postoperative infarction volume, necrosectomy volume, and posterior fossa volume), from all 49 patients who underwent posterior fossa decompression surgery for cerebellar infarction in our department from January 2012 to January 2021.

**Results:**

Thirty-five (71%) patients had a Glasgow Coma Scale (GCS) of 14–15 at admission vs. only 14 (29%) before vs. 41 (84%) following surgery. Seven (14%) patients had preventive surgery (initial GCS 14–15, preoperative GCS change ≤ 1). Only 18 (37%) patients had an mRS score of 0–3 at discharge. Estimated overall survival was 70.5% at 1 year. Interestingly, 18/20 (90%) surviving cases had a modified Rankin Scale (mRS) outcome of 0–3 (mRS 0–2: 12/20 [60%]) 1 year after surgery. Surgical timing, including preventive surgery and mass effect of the infarct, in the posterior fossa assessed semi-quantitatively (Kirollos grade) and with volumetric parameters that were not predictive of the patients' (functional) outcomes.

**Conclusion:**

Posterior fossa decompression for cerebellar infarction is a life-saving procedure, but rapid recovery of the GCS after surgery does not necessarily translate into good functional outcome. Many patients died during follow-up, but long-term mRS outcomes of 4–5 are rare. Surgery should probably aim primarily at pressure relief, and our clinical as well as volumetric data suggest that the impact of removing an infarcted tissue may be limited. It is presumably relatively safe to initially withhold surgery in cases with a GCS of 14–15.

## Introduction

Cerebellar infarctions comprise 2% of all intracranial strokes, but they have nearly twice the mortality rate of supratentorial strokes (1). A space-occupying infarction usually results in compression of the fourth ventricle and, consequently, hydrocephalus. Due to limited space in the posterior cranial fossa, a large infarction can rapidly compress the brainstem, leading to severe neurological impairment, coma, and death (1–4). Surgical decompression of the posterior cranial fossa conceptually relieves brainstem compression and, often, hydrocephalus (5, 6). Hydrocephalus treatment may also require the additional placement of an external ventricular drain. Decompression of a space-occupying cerebellar infarction can be life-saving, and it is generally accepted that surgery is warranted in neurologically deteriorating but otherwise salvageable patients (5, 7).

However, surprisingly, only few studies have evaluated indications, surgical techniques, and outcomes following surgery for cerebellar infarctions. Indeed, a recent meta-analysis describes only 283 patients (8). This is in contrast to the much more robust data concerning surgery for malignant infarction of the middle cerebral artery (9, 10). This paucity of data likely explains why many issues surrounding posterior fossa decompression surgery remain unresolved.

We have, therefore, reviewed our January 2012 to January 2020 institutional experience with surgical treatment of cerebellar infarctions. Specifically, we attempted to address three questions. First, in view of the somewhat limited body of evidence outlined above, we wanted to contribute some data to the literature regarding the time course of the patients' functional outcome and outcome predictors. Second, there is no consensus with regard to specifics of the surgical procedure (5). What is required in a technical sense for a clinically successful decompression? There are many surgical variations, and none has been unequivocally proven superior to the others. Surgical options range from craniectomy and dural expansion to actual debridement of infarcted tissues, i.e., necrosectomy. Is there a critical volume threshold for strokectomy to achieve a good outcome? Can imaging parameters help to define the appropriate surgical intervention (and predict outcomes)?

Finally, some authors have discussed surgical timing (4, 8, 11). Most will recommend surgery to restore the patient's vigilance, but the concept of preventive surgery has also attracted some attention. Kim et al. recently reported favorable results following surgery on patients with an initial GSC of nine or better (mean GCS = 12.1) who remained stable for >72 h when compared to a propensity-matched control group on which surgery was performed for clinical deterioration (11). However, many will probably disagree with these authors' treatment algorithm and use of a much higher GCS of ≤ 13 as a major criterion and cutoff for surgical decision-making. We, therefore, asked if there is a role for prophylactic surgery in cases with a GCS of 14–15.

## Methods

### Patients

The hospital's electronic database was retrospectively searched for patients who underwent posterior fossa decompression surgery for malignant cerebellar infarction in the Department of Neurosurgery, Bethel Clinic, University Hospital OWL in Bielefeld between January 2012 and January 2021. Patients with infarctions secondary to aneurysm, arteriovenous malformation or fistula treatment, or any other surgical or neurointerventional therapy were excluded. We identified *n* = 49 adult (> 18 years) cases. The study was approved by the responsible institutional review board for human research and ethics committee (Ethikkommission der Ärztekammer Westfalen-Lippe und der Westfälischen Wilhelms-Universität Münster, Germany, Az 2021-155-f-S).

### Institutional Treatment Standards

Standard practices include admission to our certified stroke unit (or ICU if ventilatory support is necessary), ultrasound of the extra- and intracranial arteries and the heart, and cardiovascular monitoring according to institutional guidelines. Treatment of thrombotic, hypertensive, diabetic, and hyperlipidic conditions is established as indicated. Patients who had a CT scan at presentation and after 24 h. A neurosurgical consult is obtained initially and whenever patients deteriorate or in case of progressive imaging findings. Per routine surgery is performed for a GCS score of 13 or lower. Decision-making is individualized in cases with good clinical conditions (i.e., GCS score of 14–15) with very large or progressive infarctions. Antiedematous or hyperosmolartherapy is not routinely prescribed. Ventricular drains are placed for symptomatic hydrocephalus at presentation or during the patient's clinical course, and if decompressive surgery alone is not felt to sufficiently restore CSF pathways. Standard surgical treatment includes necrosectomy. Additional surgical maneuvers aiming at posterior fossa decompression (craniectomy vs. craniotomy, dural expansion) are performed as deemed necessary by the attending neurosurgeon based on intraoperative findings.

### Data Collection and Variables

The clinical data, including follow-up information, were retrospectively collected from the patients' electronical charts. Pertinent clinical data were recorded, including patient demographics, comorbidities, cerebellar stroke characteristics (vascular territory, bilaterality, brainstem involvement, and additional supratentorial stroke), non-surgical treatments (intravenous thrombolysis and mechanical thrombectomy), and clinical status (initial NIHSS score (12), initial, pre- and postsurgical GCS scores (13), mRS scores (14) at discharge, and during follow-up). Stroke etiology was classified using the TOAST (trial of ORG 10172) classification (15). Thrombectomy outcomes were graded using the TICI (thrombolysis in cerebral infarction) scale as described by Higashida and Furlan (16). Follow-up information was obtained through chart reviews. The clinical status of the patients was assessed using the mRS score 3 months and 1 year following surgery, and on the last available follow-up. Our primary outcome measures were in-hospital death and mRS scores at discharge and on last follow-up. For statistical purposes, we defined a favorable outcome as mRS of 0–3; a poor outcome was defined as mRS of 4–5 or death (mRS 6).

**Figure 1:**
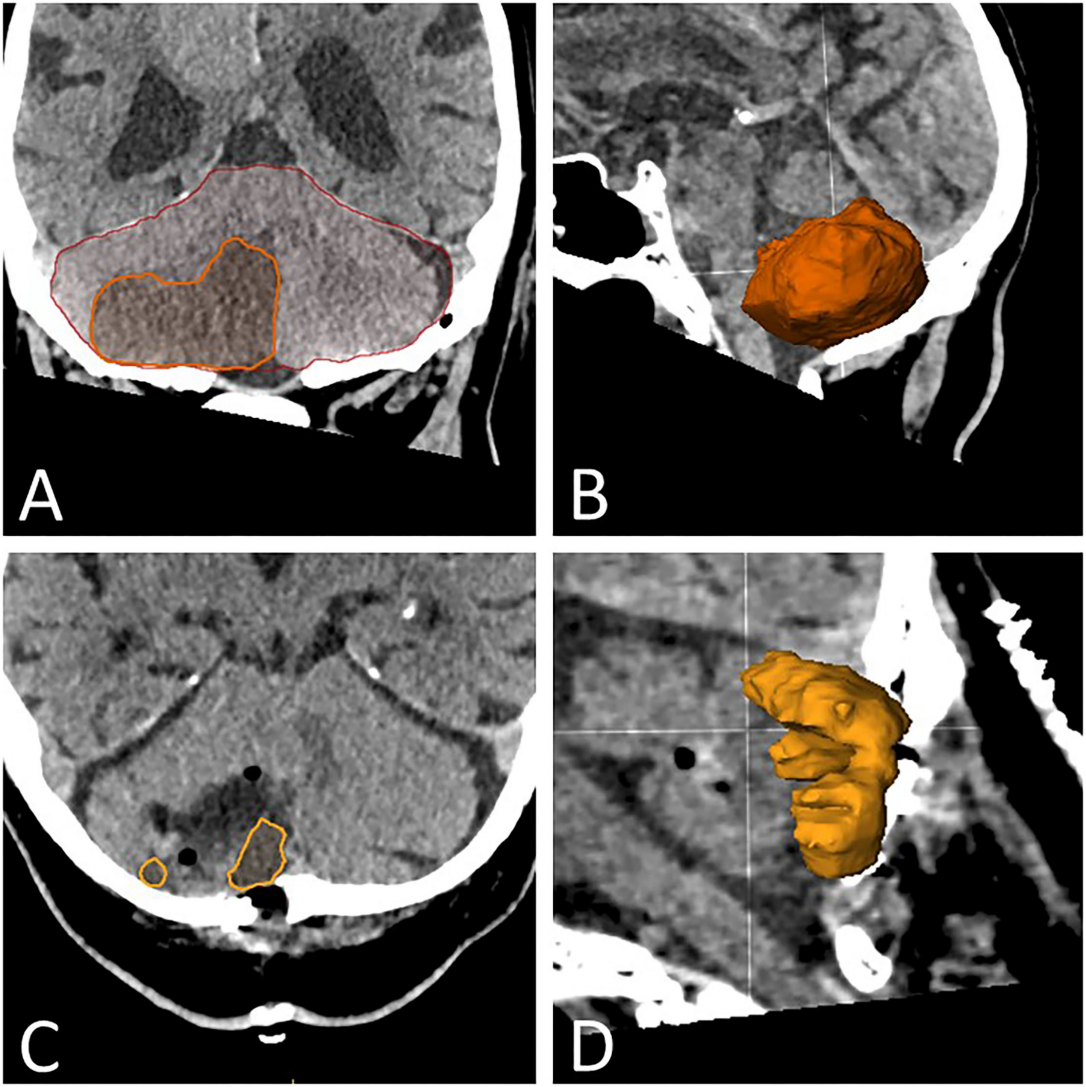
Volumetric analysis. **(A)** CT image of the cerebellum in the coronal plane depicting the segmentation of the posterior cranial fossa (red line) and of the infarcted area (yellow line). The segmentation is performed in the coronal, sagittal, and axial planes and adjusted accordingly for each slice if necessary. **(B)** Three-dimensional depiction of the cerebellar infarction for illustration purposes. **(C)** CT image of the cerebellum in the axial plane, with segmentation of residual infarction after necrosectomy (inside yellow line). **(D)** Three-dimensional depiction of the residual cerebellar infarction for illustration purposes.

We recorded the specifics of surgical treatment (including hydrocephalus management) and all surgical complications. Time to surgery was defined as the time between symptom onset and the time of incision. We used commercially available neuronavigation software (Brainlab AG, Munich, Germany) to perform computerized volumetric analyses of preoperative stroke volume, postoperative stroke volume, and total posterior fossa volume (Figure 1). Brain imaging studies immediately prior to and after surgery were used for this analysis regardless of modality (magnetic resonance imaging or computerized tomography). In cases with a second posterior fossa decompression surgery (see below), we used the first imaging study obtained after the second surgery for postoperative assessment. In addition, we analyzed the preoperative and postoperative mass effects of the infarction in the posterior fossa utilizing a semi-quantitative grading scale described by Kirollos et al. for cerebellar hemorrhage (17). Briefly, the patients are assigned to the three Kirollos grades based on the CT appearance of the fourth ventricle. Normal size and position correspond to grade 1, partial compression and obliteration to grade 2, and in grade-3 cases, the ventricle is completely obliterated.

### Statistical Analysis

The commercially available software (IBM SPSS Statistics for Windows, Version 25.0; IBM Corp., Armonk, NY, United States) and a free and open statistics package (jamovi, Version 2.0; The jamovi project) were used for all statistical analyses. Specific analyses included Fisher's exact test, the chi-square test, linear-by-linear association (Mantel-Haenszel test), and Student *t*-test for univariate analyses as indicated. Two-sided tests were conducted throughout, and *P*-values < 0.05 were considered significant. Overall survival was studied using Kaplan Meier estimates.

## Results

### Patient Characteristics

We studied a total of 49 patients whoE had surgery for a space-occupying cerebellar infarction between January 2012 and January 2021. The baseline clinical characteristics of the cohort are detailed in Table 1. The series was predominantly male (69.4%) and included only 14.3% of cases <50 yrs. of age (range 27.6–85.3 yrs.). NIHSS score at presentation was ≤4 in 19 (38.8%; “minor stroke”), 5–15 in 21 (42.9%, “moderate stroke”), 16–20 in 4 (8.2%, “moderate to severe stroke”), and ≥21 in 5 (10.2%, “severe stroke”) cases (12, 18). NIHSS score and age correlated strongly with each other (e.g., age ≥70/<70 yrs. vs. NIHSS score ≤ 4/≥ 5; *p* = 0.009). Eleven cases (22.4%) were already treated with anticoagulants, i.e., thrombocyte aggregation inhibitors in 4 (8.2%), phenprocoumon in 3 (6.1%), and NOACs in 4 (8.2%).

**Table 1:** Baseline clinical and treatment characteristics (*n* = 49).

**Demographics**	**Male/female**	**34/15 (69.4/30.6%)**
	Age	Median 70.4 (25–75% IQR: 55.5–70.4) yrs
Comorbidities	Arrhythmia	21 (42.9%)
	Smoking	8 (16.3%)
	Diabetes	15 (30.6%)
	Coronaryheartdisease	10 (20.4%)
	Hypertension	34 (69.4%)
Anticoagulation and/or platelet inhibition	No	38 (77.6%)
	Platelet inhibitors	4 (8.2%)
	Phenprocoumon	3 (6.1%)
	NOACs	4 (8.2%)
Clinical presentation	Initial NIHSS score	Median 8 (25–75% IQR: 3–8)
	GCS score at presentation	Median 15 (25–75% IQR: 13–15)
	GCS score beforesurgery	Median 11 (25–75% IQR: 8–14)
Etiology*	Large-arteryatherosclerosis	10 (20.4%)
	Cardioembolism	19 (38.8%)
	Other determinedetiology	7 (14.3%)
	Undeterminedetiology	13 (26.5%)
Treatment	IV thrombolysis	8 (16.3%)
	Thrombectomy	7 (14.3%)
	Successfulthrombectomy (TICI 2b/3)	5 (10.2%)
	Time tosurgery	Median 53 (25–75% IQR: 30–89) hrs
	Craniotomy/craniectomy	17/32 (34.7/65.3%)
	Duralexpansion	18/49 (36.7%)
	EVD	26 (52.1%)
	VP shunt	2 (4.1%)

IQR, interquartile range; yrs., years; NOAC, new oral anticoagulants; NIHSS, National Institutes of Health Stroke; GCS, Glasgow Coma Scale; *, TOAST classification. Figures do not add up to 100% because of rounding error; IV, intravenous; hrs., hours; EVD, external ventricular drain; VP, ventriculo-peritoneal.

### Medical Therapy, Surgery for Cerebellar Infarction, and Hydrocephalus Treatment

Thirty-five (71.4%) patients presented with an initial GCS score of 14–15 and 6 (12.2%) with a GCS score of <9. All the patients were admitted to a certified stroke unit and treated by a stroke neurologist. Stroke etiologies are detailed in Table 1. IV thrombolysis was performed in 8 (16.3%) and mechanical thrombectomy for basilar artery thrombosis or emboli in 7 (14.3%) cases. Mechanical recanalization was successful, i.e., TICI grade 2b/3 blood flow was restored in 5/7 (71.4%) thrombectomy cases.

**Figure 2:**
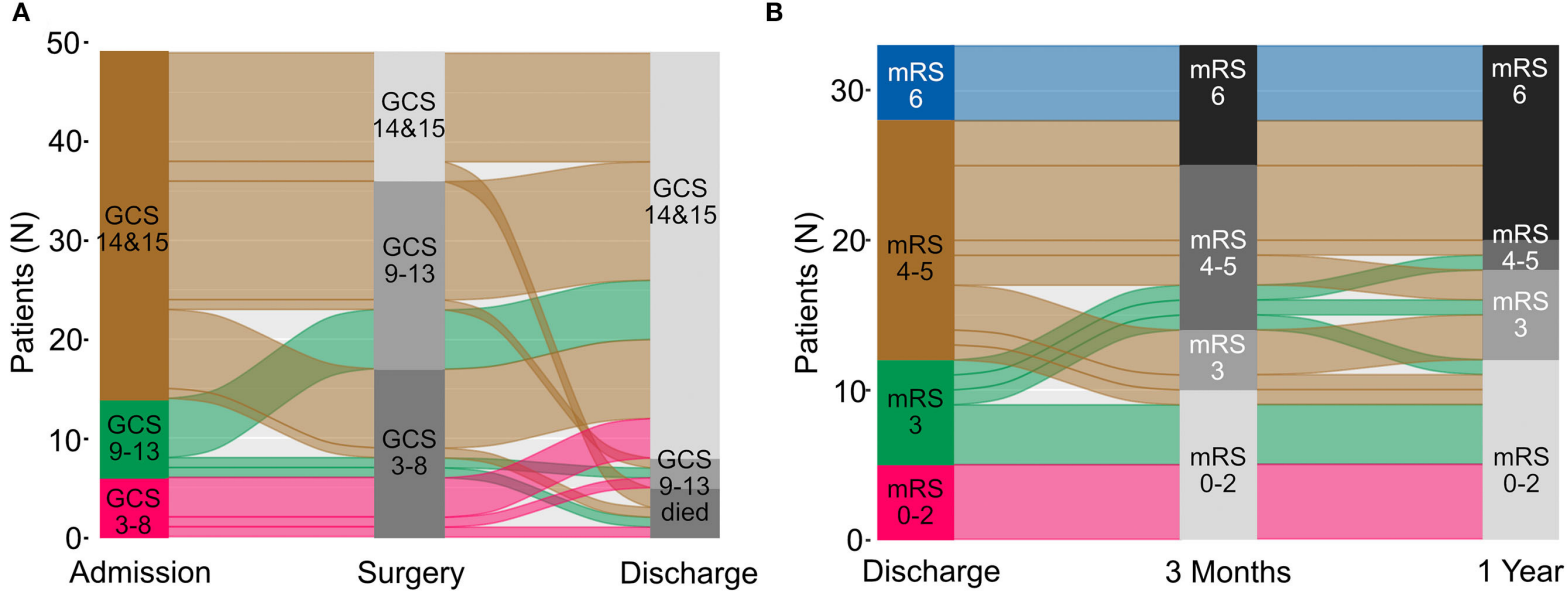
Migration plot of functional status changes from admission to discharge and during follow-up are shown as a. In **(A)** GCS scores at admission, before surgery, and at discharge are displayed for all patients. In panel **(B)** mRS scores at discharge, at 3 months and 1 year after surgery are shown in *n* = 33 patients for whom complete data were available. GCS, Glasgow Coma Scale; mRS, modified Rankin Scale.

The median time to surgery was 53 (25–75% IQR: 30–89) h. The preoperative GCS score was 14–15 in 14 (28.6%) and <9 in 17 (34.7%) patients, i.e., overall 33 (67.3%) cases deteriorated by one GCS point and 23 (46.9%) by two or more GCS points before surgery (Table 1 and Figure 2A). Surgical treatment was performed for a GCS score of 13 or lower in 35 (71.4%) cases. The remaining 14 cases (28.6%) with a GCS score of 14**–**15 had “prophylactic” surgery, i.e., decision to proceed with surgery was made to prevent substantial clinical worsening from a large infarction *vis-a-vis* only mild deterioration (GCS 14–15; *n* = 7), mild impairment (GCS 14) and failure to improve (*n* = 4), or imaging findings alone (*n* = 3).

Surgical treatment consisted of suboccipital craniectomy (*n* = 32 [65.3%]) or craniotomy (*n* = 17 [34.7%]), necrosectomy as deemed necessary for adequate decompression (and hemostasis), and duroplasty with artificial dura (*n* = 18, [36.7%]). Resection of the posterior arch of C1 was not performed. In 30 cases (61.2%), preoperative neuroimaging showed hydrocephalus (Table 2). Twenty-six of the patients (86.7%) were treated with ventricular drains either before or at the time of infarct debridement. Two of the cases later required permanent CSF drainage and had surgery for ventriculo-peritoneal shunt placement. In the remaining four hydrocephalic cases, necrosectomy was considered sufficient to restore proper CSF circulation; however, two of the patients required temporary ventricular drains later on. Two cases without hydrocephalus at presentation had ventricular drains placed for secondary hydrocephalus and CSF fistula treatment. There were no CSF diversion surgeries during follow-up; the overall shunt rate was 2/49 (4.1%; Table 1).

**Table 2:** Imaging findings.

**Vascular territories / Location**	**Unilateral PICA**	**32 (65.3%)**
	**Bilateral PICA**	**12 (24.5%)**
	**Uni-/bilateral PICA & SUCA**	**4 (8.2%)**
	**Left SUCA & right AICA**	**1 (2.0%)**
	**Additional supratentorial infarction**	**9 (18.4%)**
	**Brainstem involvement**	**12 (24.5%)**
	**Bilateral**	**14 (28.6%)**
**Hydrocephalus**		**30 (61.2%)**
**Kirollos score**	**Before surgery**	**Median 3 (25–75% IQR: 2–3)**
	**After surgery**	**Median 2 (25–75% IQR: 2–3)**
**Volumetry**	**Cerebellar infarct volume (mean)**	**64.7 ± 23.3 ml**
	**Postsurgical infarct volume (mean)**	**34.8 ± 22.4 ml**
	**Cerebellar infarct volume/ posterior fossa volume (mean)**	**33.9 ± 11.3 %**
	**Postsurgical infarct volume/posterior fossa volume (mean)**	**18.1 ± 11.3 %**

*PICA, posterior inferior cerebellar artery; SUCA, superior cerebellar artery; VA, vertebral artery; BA, basilar artery; IQR, interquartile range*.

Surgical complications included meningitis (*n* = 2 [4.1%]) and CSF fistulas (*n* = 4 [8.2%]). Three (6.1%) patients required five revision surgeries for CSF fistula repair (*n* = 4) and impaired wound healing (*n* = 1). Three other patients had a second decompression surgery (6.1%). These cases had remained in a coma, while postoperative imaging showed a crowded posterior fossa with compression of the fourth ventricle. Medical complications during the patients' hospital stay included pneumonia in *n* = 22, urinary tract infections in *n* = 6, and thromboembolic events in *n* = 1 patient(s). Seventeen cases required tracheostomy (34.7%).

### Radiology Data and Volumetric Analysis

Imaging findings are detailed in Table 2. Neuroimaging revealed brainstem involvement in eight cases (16.3%). Mass effect was assessed using the classification described by Kirollos et al. for cerebellar hemorrhage (17, 19). Thirty-two (65.3%) cases had grade 3 (obliterated fourth ventricle, anterior displacement), 16 (32.7%) had grade 2 (distorted fourth ventricle), and one had grade 1 (normal size and configuration of the fourth ventricle) CT findings. The postoperative scans showed improved Kirollos grades in 17 (34.7%), stable grades in 30 (61.2%), and worsened grades in two cases. There were no significant correlations between the preoperative Kirollos grade and the patients' GCS at presentation and before surgery (GCS 14–15 vs. ≤13), and postoperative Kirollos grade and discharge GCS (GCS 14–15 vs. ≤13).

The volumetric analysis allowed for a more quantitative evaluation of the mass effect of the cerebellar infarction and its surgical relief (Table 2). The mean size of the cerebellar infarcts was 64.7 ± 23.3 ml, i.e., 33.9 ± 11.3% of the overall posterior fossa volume. The typical postoperative infarction volume following necrosectomy was approximately half as large. Mean (relative) infarct size was correlated strongly with GCS score at the time of surgery (cerebellar infarct volume, GCS 14**-**15 vs. ≤13: 51.2 ± 13.3 vs. 70.1 ± 24.4 ml, *p* = 0.001 and cerebellar infarct volume/posterior fossa volume: 28.3 ± 8.8 vs. 36.1 ± 11.6 %, *p* = 0.016). No such correlations were seen for GCS score at presentation and discharge GCS score. Preoperative volumetric measurements also did not correlate with GCS deterioration before surgery. We found no statistically significant correlation between discharge GCS score and postoperative volumetric data. There were significant correlations between NIHSS score and preoperative absolute (NIHSS score ≤4, 5–15, ≥16: 65.2 ± 17.5, 57.1 ± 20.5, and 81.4 ± 32.4 ml; *p* = 0.029) and relative infarction volumes (cerebellar infarct volume/posterior fossa volume, NIHSS score ≤4, 5–15, ≥16: 32.6 ± 8.9, 31.4 ± 10.5, and 42.6 ± 14.5%; *p* = 0.034).

### Patient Outcomes

In-hospital mortality was 5 (10.2%). Three patients died after the limitation of treatment *vis-à-vis* persisting coma according to the patient's will and consultation with her or his relatives. Two patients succumbed to complications of an underlying disease that had caused the initial cerebellar stroke (septic endocarditis: *n* = 1, metachronous malignant MCA infarction caused by atrial fibrillation: *n* = 1). Forty-one of the remaining 44 (93.2%) patients had a discharge GCS of 14–15 vs. 14/49 (28.6%) at the time of surgical infarct debridement, i.e., surgery restored the patients' vigilance in the majority of our cases (Figure 2).

**Figure 3:**
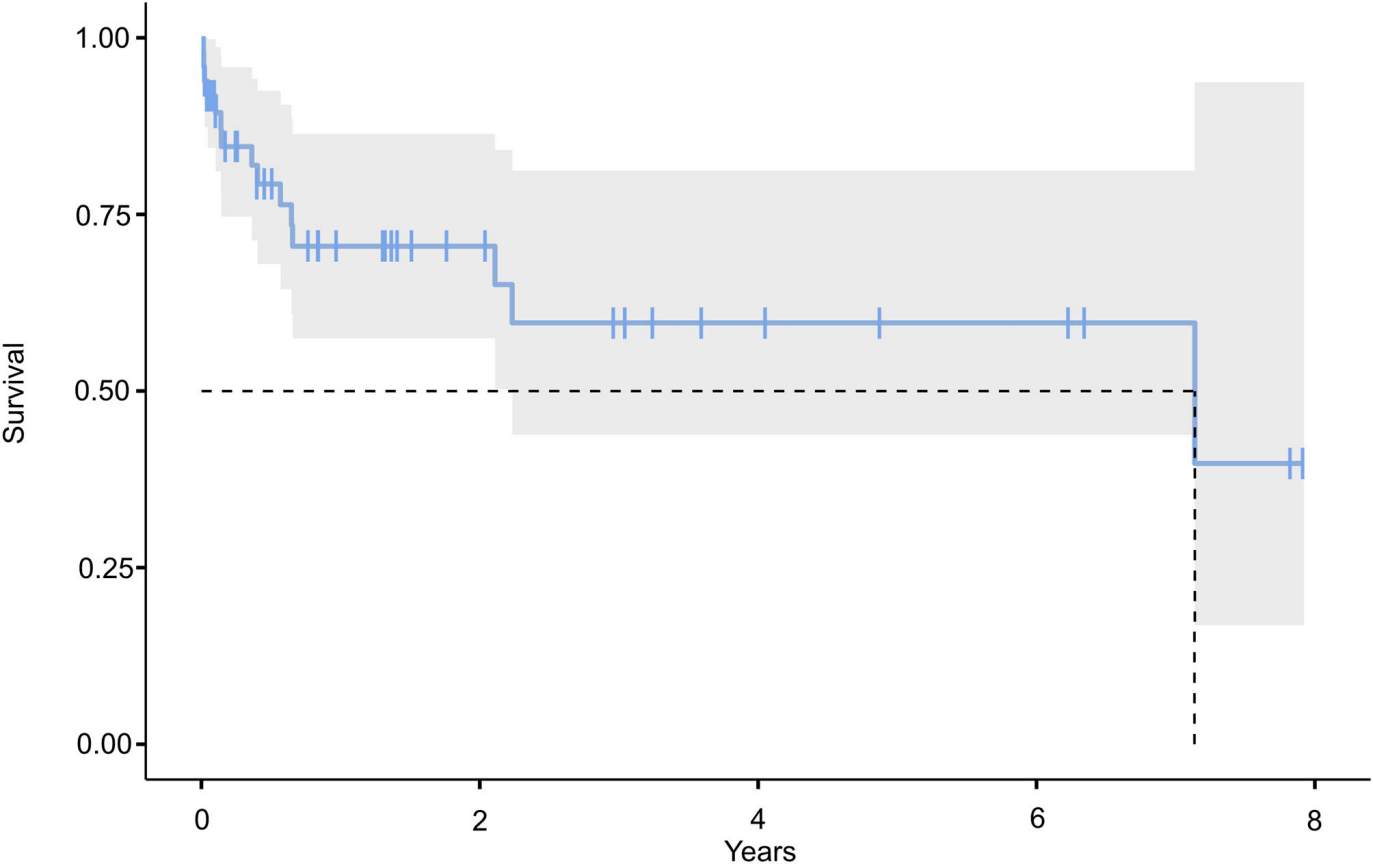
Kaplan Meier estimation of overall survival.

The functional and survival outcomes following surgery in this cohort were limited (Figure 2 and Table 3). Only seven (14.3%) patients had an mRS score of 0–2 at discharge (mRS 0–3: 18, 36.7%). Fifteen patients were followed until death, and the median follow-up was 13.9 (25–75% IQR: 2.8–37.7) months in the remainder. mRS 0–2 and mRS 0–3 outcomes were seen in 17 (34.7%) and 27 (55.1%) of the cases on last follow-up (Figure 2). 3-month follow-ups were available for 41 patients, and 1-year follow-ups in 33 cases. mRS outcome improved somewhat over time. The percentage of patients with mRS 0–2 and 0–3 outcomes increased to 29.3 and 53.7% at 3 months, and 36.4 and 54.6% at 1 year; 18/20 (90%) surviving cases had an mRS 0–3 outcome at 1 year (mRS 0-2: 12/20 [60%]). Conversely, the percentage of cases with an mRS 4–5 outcome decreased from 53 (discharge) to 31.7% (3 months) and 6.1% at 1 year. Figure 2B shows that most patients with an initial mRS 4–5 outcome either improved or died during follow-up. Overall survival was only 70.5% at 1 year, and the Kaplan Meier (Figure 3) estimate of median overall survival was 86.8 (95% CI: 0–189.3) months.

**Table 3:** Functional outcomes after surgery over time.

**mRS**	**0**	**1**	**2**	**3**	**4**	**5**	**6**
Discharge (*N* = 49)	0	3 (6.1%)	4 (8.2%)	11 (22.4%)	13 (26.5%)	13 (26.5%)	5 (10.2%)
3 months (*N* = 41)	1 (2.4%)	4 (9.8%)	7 (17.1%)	8 (19.5%)	10 (24.4%)	3 (7.3%)	8 (19.5%)
1 yr (*N* = 33)	2 (6.1%)	6 (18.2%)	4 (12.1%)	6 (18.2%)	2 (6.1%)	0	13 (39.4%)
Last f-UP (*N* = 49)	2 (4.1%)	9 (18.4%)	6 (12.2%)	10 (20.4%)	8 (16.3%)	1 (2.0%)	13 (26.5%)

*mRS, modified Rankin Scale; yr, year; f-up, follow-up*.

### Outcome Predictors

We tested various patient and infarct characteristics and treatment variables as possible outcome predictors (Table 4). A medical history of anticoagulation was the only significant predictor of in-hospital mortality largely reflecting an association with phenprocoumon (but not NOAC) treatment (*p* < 0.001). All three patients who had a cerebellar infarction while under medication with phenprocoumon died (*p* = 0.001). There was also a borderline association with initial NIHSS score.

**Table 4:** Patient, infarct and treatment characteristics as predictors of in-hospital death, and functional outcomes at discharge and last follow-up.

			**In-hospital death**			**mRSat discharge**			**mRS at last f-up**		
			**No**	**Yes**	**P**	**0–3**	**4–6**	**P**	**0–3**	**4–6**	**P**
Age	≤ 70 yrs. (median)	24 (49.0%)	23 (95.8%)	1 (4.2%)	0.349	12 (50.0%)	12 (50.0%)	0.059	18 (75.0%)	6 (25.0%)	0.006
	>70 yrs.	25 (51.0%)	21 (84.0%)	4 (16.0%)		6 (24.0%)	19 (76.0%)		9 (36.0%)	16 (64.0%)	
Sex	Female	15 (30.6%)	13 (86.7%)	2 (13.3%)	0.635	6 (40.0%)	9 (60.0%)	0.753	7 (46.7%)	8 (53.3%)	0.430
	Male	34 (69.4%)	31 (91.2%)	3 (8.8%)		12 (35.3%)	22 (64.7%)		20 (58.8%)	14 (41.2%)	
Arrhythmia	Yes	21 (42.9%)	18 (85.7%)	3 (14.3%)	0.639	8 (38.1%)	13 (61.9%)	0.864	12 (57.1%)	9 (42.9%)	0.136
	No	28 (57.1%)	26 (92.9%)	2 (7.1%)		10 (35.7%)	18 (64.3%)		10 (35.7%)	18 (64.3%)	
Smoking	Yes	8 (16.3%)	8 (100%)	0 (0%)	0.575	4 (50%)	4 (50%)	0.443	6 (75.0%)	2 (25.0%)	0.269
	No	41 (83.7%)	36 (87.8%)	5 (12.2%)		14 (34.1%)	27 (65.9%)		21 (51.2%)	20 (48.8%)	
Diabetes	Yes	15 (30.6%)	13 (86.7%)	2 (13.3%)	0.635	4 (26.7%)	11 (73.3%)	0.521	6 (40.0%)	9 (60.0%)	0.158
	No	34 (69.4%)	31 (91.2%)	3 (8.8%)		14 (42.2%)	20 (58.8%)		21 (61.8%)	13 (38.2%)	
Coronaryheartdisease	Yes	11 (22.4%)	11 (100%)	0 (0%)	0.574	4 (36.4%)	7 (63.6%)	1.000	8 (72.7%)	3 (27.3%)	0.303
	No	38 (77.6%)	33 (86.8%)	5 (13.2%)		14 (36.8%)	24 (63.2%)		19 (50.0%)	19 (50.0%)	
Hypertension	Yes	34 (69.4%)	30 (88.2%)	4 (11.8%)	1.000	13 (38.2%)	21 (61.8%)	1.000	17 (50.0%)	17 (50.0%)	0.358
	No	15 (30.6%)	14 (93.3%)	1 (6.7%)		5 (33.3%)	10 (66.7%)		10 (66.7%)	5 (33.3%)	
Anticoagulation or platelet inhibition	no	38 (77.6%)	37 (97.4%)	1 (2.6%)	<0.001	17 (44.7%)	21 (55.3%)	0.148	23 (60.5%)	15 (39.5%)	0.239
	Platelet inhibitors	4 (8.2%)	3 (75.0%)	1 (25.0%)		0 (0%)	4 (100%)		2 (50.0%)	2 (50.0%)	
	Phenprocoumon	3 (6.1%)	0 (0%)	3 (100%)		0 (0%)	3 (%)		0 (0%)	3 (%)	
	NOACs	4 (8.2%)	4 (0%)	0 (0%)		1 (25.0%)	3 (75.0%)		2 (50.0%)	2 (50.0%)	
NIHSS score at presentation	≤ 4	19 (38.8%)	19 (100%)	0 (0%)	0.053	13 (68.4%)	6 (31.6%)	0.003	15 (78.9%)	4 (21.1%)	0.032
	5**–**15	21 (42.9%)	18 (85.7%)	3 (14.3%)		3 (14.3%)	18 (85.7%)		8 (38.1%)	13 (61.9%)	
	≥16	9 (18.4)	7 (77.8%)	2 (22.2%)		2 (22.2%)	7 (77.8%)		4 (44.4%)	5 (55.6%)	
GCS score at presentation	14**–**15	35 (71.4%)	32 (91.4%)	3 (8.6%)	0.616	15 (42.9%)	20 (57.1%)	0.202	20 (57.1%)	15 (42.9%)	0.650
	≤ 13	14 (28.6%)	12 (85.7%)	2 (14.3%)		3 (21.4%)	11 (78.5%)		7 (50.0%)	7 (50.0%)	
GCS score at presentation	14**–**15	35 (71.4%)	32 (91.4%)	3 (8.6%)	0.810	15 (42.9%)	20 (57.1%)	0.270	20 (57.1%)	15 (42.9%)	0.902
	9**–**13	8 (16.3%)	7 (87.5%)	1 (12.5%)		7 (87.5%)	1 (12.5%)		4 (50.0%)	4 (50.0%)	
	3**–**8	6 (12.2%)	5 (83.3%)	1 (16.7%)		4 (66.7%)	2 (33.3%)		3 (50.0%)	3 (50.0%)	
GCSscore before surgery	14**–**15	14 (28.6%)	12 (85.7%)	2 (14.3%)	0.616	6 (42.9%)	8 (57.1%)	0.574	9 (64.3%)	5 (35.7%)	0.530
	≤ 13	35 (71.4%)	32 (71.4%)	3 (8.6%)		12 (34.3%)	23 (65.7%)		18 (51.4%)	17 (48.5%)	
GCS score before surgery	14**–**15	14 (28.6%)	12 (85.7%)	2 (14.3%)	0.683	6 (42.9%)	8 (57.1%)	0.721	9 (64.3%)	5 (35.7%)	0.705
	9**–**13	18 (36.7%)	18 (100%)	0 (0%)		7 (38.9%)	11 (61.1%)		9 (50.0%)	9 (50.0%)	
	3**–**8	17 (34.7%)	14 (82.4%)	3 (17.6%)		5 (29.4%)	12 (70.6%)		9 (52.9%)	8 (47.1%)	
GCS score deterioration before surgery	Yes	33 (67.3%)	30 (90.9%)	3 (9.1%)	1.000	13 (39.4%)	20 (60.6%)	0.579	16 (48.5%)	17 (51.5%)	0.181
	No	16 (32.7%)	14 (87.5%)	2 (12.5%)		5 (31.3%)	11 (68.8%)		11 (68.8%)	5 (31.3%)	
Hydrocephalus	Yes	30 (61.2%)	27 (90.0%)	3 (10.0%)	1.000	16 (53.3%)	14 (46.7%)	0.003	18 (60.0%)	12 (40.0%)	0.386
	No	19 (38.8%)	17 (89.5%)	2 (10.5%)		2 (10.5%)	17 (89.5%)		9 (47.4%)	10 (52.6%)	
Brainstem involvement	Yes	12 (24.5%)	10 (83.3%)	2 (16.7%)	0.584	1 (8.3%)	11 (91.7%)	0.036	4 (33.3%)	8 (66.7%)	0.104
	No	37 (75.5%)	34 (91.9%)	3 (8.1%)		17 (45.9%)	20 (54.1%)		23 (62.2%)	14 (37.8%)	
Bilateral infarcts	Yes	14 (28.6%)	14 (100%)	0 (0%)	0.303	5 (35.7%)	9 (64.3%)	0.925	9 (64.3%)	5 (35.7%)	0.414
	No	35 (71.4%)	30 (85.7%)	5 (14.3%)		13 (37.1%)	22 (62.9%)		18 (51.4%)	17 (48.6%)	
Supratentorial infarcts	Yes	9 (18.4%)	8 (88.9%)	1 (11.1%)	1.000	3 (33.3%)	6 (66.7%)	1.000	6 (66.7%)	3 (33.3%)	0.48
	No	40 (81.6%)	36 (90.0%)	4 (10.0%)		15 (37.5%)	25 (62.5%)		21 (52.5%)	19 (47.5%)	
Etiology^*^	Large-arteryatherosclerosis	10 (20.4%)	9 (90.0%)	1 (10.0%)	0.974	2 (20.0%)	8 (80.0%)	0.219	3 (30.0%)	7 (70.0%)	0.311
	Cardioembolism	19 (38.8%)	17 (89.5%)	2 (10.5%)		10 (52.6%)	9 (47.4%)		11 (57.9%)	8 (42.1%)	
	Other determined etiology	7 (14.3%)	6 (85.7%)	1 (14.3%)		3 (42.9%)	4 (57.1%)		5 (71.4%)	2 (28.6%)	
	Undetermined etiology	13 (26.5%)	12 (92.3.8%)	1 (7.7%)		3 (23.1%)	10 (76.9%)		8 (61.5%)	5 (38.5%)	
Previous IV thrombolysis	Yes	8 (16.3%)	7 (87.5%)	1 (12.5%)	1.000	1 (12.5%)	7 (87.5%)	0.229	4 (50.0%)	4 (50.0%)	0.751
	No	41 (83.7%)	37 (90.2%)	4 (9.8%)		17 (41.5%)	24 (58.5%)		23 (56.1%)	18 (43.9%)	
Previous thrombectomy	Yes	7 (14.3%)	6 (85.7%)	1 (14.3%)	0.554	0 (0%)	7 (100%)	0.038	1 (14.3%)	6 (85.7%)	0.036
	No	42 (85.7%)	38 (90.5%)	4 (9.5%)		18 (42.9%)	24 (57.1%)		26 (61.9%)	16 (38.1%)	
Successful thrombectomy^**^	Yes	5 (10.2%)	4 (80.0%)	1 (20.0%)	0.430	0 (0%)	5 (100%)	0.143	1 (20.0%)	4 (80.0%)	0.160
	No	44 (89.8%)	40 (90.9%)	4 (9.1%)		18 (40.9%)	26 (59.1%)		26 (59.1%)	18 (40.9%)	
Preoperative Kirollos grade	I	1 (2.0%)	1 (100%)	0 (0%)	0.101	0 (0%)	1 (100%)	0.143	1 (100%)	0 (0%)	0.965
	II	16 (32.7%)	16 (100%)	0 (0%)		4 (25.0%)	12 (75.0%)		8 (50.0%)	8 (50.0%)	
	III	32 (65.3%)	27 (84.4%)	5 (15.6%)		14 (43.8%)	18 (56.3%)		18 (56.3%)	14 (43.8%)	
Postoperative Kirollos grade	I	6 (12.2%)	6 (100%)	0 (0%)	0.273	1 (16.7%)	5 (83.3%)	0.229	3 (50.0%)	3 (50.0%)	0.585
	II	23 (46.9%)	21 (91.3%)	2 (8.7%)		7 (30.4%)	16 (69.4%)		12 (52.2%)	11 (47.8%)	
	III	20 (40.8%)	17 (85.0%)	3 (15.0%)		10 (50.0%)	10 (50.0%)		12 (60.0%)	8 (40.0%)	
Cerebellar infarct volume	≥64.7 ml (mean)	20 (40.8%)	16 (80.0%)	4 (20.0%)	0.144	5 (25.0%)	15 (75.0%)	0.230	10 (50.0%)	10 (50.0%)	0.551
	<64.7 ml	29(59.2%)	28 (96.6%)	1 (3.4%)		13 (44.8%)	16 (55.2%)		17 (58.6%)	12 (41.4%)	
Post-surgical infarct volume	≥34.8 ml (mean)	20 (40.8%)	16 (80.0%)	4 (20.0%)	0.144	6 (30.0%)	14 (70.0%)	0.417	11 (55.0%)	9 (45.0%)	0.990
	<34.8 ml	29 (59.2%)	28 (96.6%)	1 (3.4%)		12 (41.4%)	17 (58.6%)		16 (55.2%)	13 (44.8%)	
Cerebellar infarct volume/ posterior fossa volume	≥33.9% (mean)	23 (46.9%)	20 (87.0%)	3 (13.0%)	0.655	6 (23.1%)	17 (76.9%)	0.146	12 (52.2%)	11 (47.8%)	0.698
	<33.9%	26 (53.1%)	24 (92.3%)	2 (7.7%)		12 (46.2%)	14 (53.8%)		15 (57.7%)	11 (42.3%)	
Postsurgical infarct volume/ posterior fossa volume	≥18.1% (mean)	20(40.8%)	16 (80.0%)	4 (20.0%)	0.144	7 (35.0%)	13 (65.0%)	0.834	11 (55.0%)	9 (45.0%)	0.990
	<18.1%	29(59.2%)	28 (96.6%)	1 (3.4%)		11 (37.9%)	18 (62.1%)		16 (55.2%)	13 (44.8%)	
Time to surgery	≤ 48 hrs.	31 (63.3%)	28 (90.3%)	3 (9.7%)	1.000	10 (32.3%)	21 (67.7%)	0.394	17 (54.8%)	14 (45.2%)	0.961
	>48 hrs.	18 (36.7%)	16 (88.9%)	2 (11.1%)		8 (44.4%)	10 (55.5%)		10 (55.6%)	8 (44.4%)	
Craniectomy	Yes	32 (65.3%)	29 (90.6%)	3 (9.4%)	1.000	10 (31.3%)	22 (68.8%)	0.275	17 (53.1%)	15 (46.9%)	0.703
	No	17 (34.7%)	15 (88.2%)	2 (11.8%)		8 (47.1%)	9 (52.9%)		10 (58.8%)	7 (41.2%)	
Dural expansion	Yes	18 (36.7%)	17 (94.4%)	1 (5.6%)	0.639	6 (33.3%)	12 (66.7%)	0.707	11 (61.1%)	7 (38.9%)	0.519
	No	31 (63.3%)	27 (87.1%)	4 (12.9%)		12 (38.7%)	19 (61.3%)		16 (51.6%)	15 (48.4%)	
Preoperative EVD	Yes	26 (53.1%)	23 (88.5%)	3 (11.5%)	1.000	13 (50.0%)	13 (50.0%)	0.074	16 (61.5%)	10 (38.5%)	0.336
	No	23 (46.9%)	21 (91.3%)	2 (8.7%)		5 (21.7%)	18 (78.3%)		11 (47.8%)	12 (52.2%)	

*mRS, modified Rankin Scale; f-up, follow-up; yrs., years; NIHSS, National Institutes of Health Stroke Scale; GCS, Glasgow Coma Scale;*

* *TOAST classification. Figures do not add up to 100% because of rounding error;*

** *TICI grade 2b/3, VA/BA, vertebral/basilar artery; IV, intravenous; hrs., hours*.

Favorable discharge mRS scores correlated with low initial NIHSS score and presence of hydrocephalus. There was a borderline association with younger age. Younger age became a significant outcome predictor at the last follow-up, while the impact of NIHSS score was weaker although still significant. Hydrocephalus no longer predicted the outcome at long-term follow-up. Previous thrombectomy was a negative prognostic factor both at discharge and at last follow-up. No patient with thrombectomy had an mRS score of 0–3 at discharge. The figures did not change much, but statistical significance was lost when analyzing for successful (i.e., TICI grade 2b/3) rather than all thrombectomies. There was a statistically significant correlation between brainstem involvement and an mRS 0–3 outcome at discharge or at last follow-up, but no patient with brainstem involvement had an mRS score of 0–2 at discharge.

All other patient and infarct characteristics as well as comorbidities were not prognostic. Neither GCS score at presentation nor preoperative GCS score was correlated significantly with the patients' functional outcomes. Importantly, we obtained no evidence that prophylactic operations (defined as surgery on a patient with a GCS of 14–15 with the intention to prevent neurological worsening or bad functional outcome) were helpful. The patients' functional outcomes also did not vary significantly with surgical timing (i.e., time to surgery). Neither the preoperative and postoperative Kirollos scores nor the absolute cerebellar infarct volume or the percentage of the overall posterior fossa volume before and after surgery varied significantly with mRS outcome. These latter results did not change after correction for craniotomy vs. craniectomy.

## Discussion

Large cerebellar infarctions cause a significant mass effect on the posterior fossa, which may result in compression of the posterior fossa with consecutive hydrocephalus and, importantly, brainstem compression (1, 2). Historically, mortality with conservative treatment has been very high. Mortality rates in the range of 40–80% have been reported in the older literature (20, 21). Somewhat earlier than for malignant supratentorial infarctions, this has led many neurosurgeons to adopt a policy of recommending surgery for symptomatic large space-occupying cerebellar infarctions.

However, many aspects of surgical treatment for posterior fossa infarctions still remain to be clarified. To a large degree, this reflects the surprisingly small number of pertinent studies that can be found in the literature and the limited number of patients included. This review of our institutional experience with the surgical treatment of cerebellar infarcts was prompted by this somewhat less than optimal database and by two issues encountered in everyday clinical practice, i.e., how and when to operate.

Surgical strategies vary considerably, and options range from decompressive craniectomy and dural expansion to craniotomy and necrosectomy with primary dural closure and replacement of bone flap. At this point, the literature certainly does not allow for delineation of operative standards. However, all cases undergo preoperative CT or MR imaging, and virtually all patients have postoperative imaging studies as well. We, therefore, investigated if imaging markers might help to predict patient outcomes and to define what constitutes an appropriate surgical procedure. We were somewhat surprised that neither a semi-quantitative (the Kirollos) score that measures the “crowding” of the posterior fossa nor volumetric measurements of the postoperative infarct predicted the patients' functional outcome. The surgeries restored the vigilance of most of our cases regardless of the postoperative imaging findings. One could tentatively conclude from these data that the primary goal of posterior fossa decompression surgery is simply the relief of (perhaps locoregional) intracranial pressure. We were unable to show that additional measures, i.e., aggressive debridement of ischemic tissues and restoration of near-normal anatomical relationships in the posterior fossa as evidenced by postoperative imaging resulted in relatively better vigilance and functional outcomes.

Some have argued that ischemic tissues cause edema and secondary parenchymal damage, likely mediated through neuroinflammatory pathways (22–24). Debridement of infarcted tissues will conceptually counteract these pathomechanisms, which would favor necrosectomy over decompressive craniectomy and dural expansion for cerebellar infarcts. While our data do not substantiate this concept, it is quite conceivable that the heterogeneous nature and limited size of our cohort simply did not allow for delineation of such effects. The actual removal of a compressive lesion i.e., strokectomy, seems to hold a greater potential of reestablishing posterior fossa CSF pathways than dural expansion alone. Lee et al. report an 8/50 (16.0%) shunt rate in their series of cases undergoing decompression only (19), compared to 2/49 (4.1%) in the present cohort. Dural expansion may also carry a higher risk of incurring a CSF fistula than primary dural closure followed by replacement of the bone flap (25). In lieu of robust and more extensive comparative data, it is probably wise to base one's decision to remove an ischemic cerebellar tissue predominantly on the latter points rather than the concept of removing a lesion capable of inducing some kind of secondary damage.

It is somewhat disappointing that the impact of various surgical maneuvers on the patients' functional outcome, beyond restoring vigilance, seems limited. However, in some ways, this parallels the experience with surgery for supratentorial infarctions. Decompressive hemicraniectomies for malignant space-occupying supratentorial infarcts prevent coma and death, but functional outcomes largely reflect the location and extent of infarctions as well as patient characteristics, such as age, rather than specifics of the surgery beyond pressure relief (10, 26). There are also similarities to the results seen after operations for cerebellar hemorrhage. A recent meta-analysis used propensity score-matched cohorts of cases with cerebellar hemorrhage to compare outcomes after conservative vs. surgical treatment. This analysis failed to show that removing a mass lesion carries better functional results (27). Rather, this study suggested that surgery for “small” hematomas (≤12 ml) was associated with even worse functional outcomes, and that cases with larger hemorrhages (≥15 ml) benefitted from surgery with respect to survival but not functional outcome.

The experience with surgery for malignant supratentorial infarctions has suggested a role for early surgical intervention, i.e., operations should ideally be performed before patients deteriorate neurologically (9). There is also some evidence to suggest that such strategies might limit the extent of the infarction to some degree because surgical decompression will restore blood flow in brain areas otherwise suffering from secondary ischemia due to increased intracranial pressure (28). It should, therefore, not come as a surprise that similar concepts have also been applied to the surgical treatment of cerebellar infarctions. However, we found no evidence in our cohort that favored proactive surgery. Neither time to surgery nor GCS score deterioration before surgery nor preoperative GCS score was correlated significantly with the patients' vigilance and functional outcomes. Evidently, these results are based on relatively few cases taken to the operating room with a truly low GCS score (GCS 3: *n* = 3, GCS 1: *n* = 5) and against the background of a policy that routinely advocates surgery in cases with a GCS ≤13. Nevertheless, we feel that our experience suggests that it is relatively safe to withhold surgery in cases with large infarctions but a GCS of 14–15.

Others have reported seemingly contradictory results. Kim et al. have compared prophylactic surgery with surgery for neurological deterioration in cases with large cerebellar infarctions (11). These authors described better outcomes following early surgery but used somewhat unusual criteria for surgical decision-making and for definition of prophylactic surgery, which rendered any comparisons very difficult. Similar to us, most neurosurgeons will operate for deterioration and for a stable GCS ≤ 13. Conversely, Kim et al. used GCS 9 as cutoff and continued with conservative management *vis-à-vis* a stable GCS of >9 (11).

Despite surgical management, malignant cerebellar infarction has a high mortality rate. Of note, all the in-hospital deaths in our cohort were due to dismal neurological conditions following cerebellar stroke or an underlying disease that had caused the infarction. Published mortality rates are in the 20–25% range (11, 19, 29, 30). Kim et al. reported unusually good mortality figures (4%) in their series of 28 surgical cases undergoing what these authors defined as preventive surgery (11). We observed a 10.2% mortality rate at discharge. Complications were not uncommon, with 12.2% (6/49) of our patients requiring revision surgery, including four cases with CSF fistulas (8.2%), and two cases (4.1%) were treated with antibiotics for meningitis. Similar figures have also been reported by others (revision surgeries: 7–12% (11, 29), surgical site infections: 4–6% (8, 29), CSF fistulas: 4–9% (8, 11, 29).

Functional outcomes after posterior fossa decompression surgery are generally not good either. The more recent studies report early mRS 0–3 outcomes in only 24–34% (19, 29) (cf. 36.7% in our cohort). It seems noteworthy that these outcome figures are by no means stable, which is likely a testament to the underlying disease(s) and severe neurological conditions caused by the infarction, i.e., mortality figures increase over time. The Kaplan-Meier mortality estimate for our series was 29.5% at 1 year. Conversely, a substantial proportion of patients continued to improve over time. Most patients with an mRS 4–5 outcome either improved or died during follow-up. At 1 year, 90% of our surviving patients had an mRS 0–3 outcome. Studies with follow-up have reported less detailed but largely similar data (19, 30).

Prognostic factors other than surgical strategies and surgical timing issues have also been discussed in the literature in some detail (30–34). However, the generally limited size of the respective cohorts renders any firm conclusion difficult and may explain why different authors have reported different results. Preoperative GSC score (often GCS <9 or “coma” is used as the cutoff), NIHSS score, age, brainstem and bilateral infarctions, basilar artery thrombosis, and infarct volume have all been reported to be correlated with patient outcome. Our results confirm some of these findings. Previous mechanical thrombectomy was associated with adverse functional outcomes in our series. This likely reflects the severity of the underlying condition in particular in cases without successful recanalization. Statistical significance of the finding was lost when analyzing successful rather than all thrombectomies. In-hospital death was correlated with phenprocoumon medication. Treatment with phenprocoumon may well be a surrogate parameter for an adverse overall medical and/or neurological condition. Of note, no such effects were seen in patients undergoing treatment with NOACs. Hydrocephalus was associated with favorable early outcomes, which may simply reflect that hydrocephalus treatment (i.e., temporary or, if necessary, permanent CSF drainage) is relatively straightforward and patients usually improve quickly and often completely, while recovery from neurological impairment caused by an infarction takes more time, and many are left with residual deficits. The prognostic role of age at last follow-up could in part be explained by the association of older age with relevant comorbidities and the generally negative effect of older age on neurological recovery.

Our study has significant limitations. The patients were treated over a 10-year period and analyzed only retrospectively. We report 49 cases. This figure is not very large (even though it compares favorably with the recent literature (11, 19, 29, 30). Follow-up data were incomplete. There are technical limitations to the volumetric analyses. Neuroimaging protocols varied over time and could include MR as well as CCT studies, which might have affected adversely the precision of the volumetric analyses.

Posterior fossa decompression proved an effective and sometimes life-saving treatment for malignant cerebellar infarctions, but rapid recovery of GCS after surgery did not necessarily translate into good functional outcome. Many patients were left considerably disabled early on following their operation, and mortality, over time, was significant. Survivors, however, often improved significantly, and only few had an mRS 4–5 long-term outcome. In this series, functional outcomes did not vary with specifics of the operation. These data suggest that pressure relief rather than restoration of anatomical relationships in the posterior fossa should be aimed at, and our clinical data and volumetric analyses suggest that the additional impact of necrosectomy is limited. We found no evidence in favor of preventive surgery based on imaging rather than clinical criteria. It is presumably relatively safe to initially withhold surgery with a GCS score of 14–15. However, a randomized controlled trial would clearly be required to properly address this issue.

## Data Availability Statement

The raw data supporting the conclusions of this article will be made available by the authors, without undue reservation.

## Ethics Statement

The studies involving human participants were reviewed and approved by Ethik-Kommission der Ärztekammer Westfalen-Lippe und der Westfälischen Wilhelms-Universität Münster. Written informed consent for participation was not required for this study in accordance with the national legislation and the institutional requirements.

## Author Contributions

EG contributed to development of methods, data collection, volumetry, manuscript writing, and manuscript review. SR contributed to development of methods and manuscript review. IG contributed to data collection and manuscript review. W-RS contributed to manuscript review. AG contributed to development of methods, figures, volumetry, and manuscript review. MS contributed to development of methods, statistical analysis, manuscript writing, and manuscript review. All authors contributed to the article and approved the submitted version.

## Funding

This study was funded by the Department of Neurosurgery, Evangelisches Klinikum Bethel, University Hospital OWL, University Bielefeld, Campus Bielefeld-Bethel, Bielefeld, Germany.

## Conflict of Interest

The authors declare that the research was conducted in the absence of any commercial or financial relationships that could be construed as a potential conflict of interest.

## Publisher's Note

All claims expressed in this article are solely those of the authors and do not necessarily represent those of their affiliated organizations, or those of the publisher, the editors and the reviewers. Any product that may be evaluated in this article, or claim that may be made by its manufacturer, is not guaranteed or endorsed by the publisher.
